# Supplementary material for: Motivators and barriers to the acceptability of a human milk bank among Malaysians
Source: PLoS One. 2024 Mar 4;19(3):e0299308. doi: 10.1371/journal.pone.0299308 (PMC10911625; doi:10.1371/journal.pone.0299308)
Supplement: S1 Checklist — (PDF) [file pone.0299308.s001.pdf]

STROBE Statement—checklist of items that should be included in reports of observational studies

	Item No.	Recommendation	Page No.	Relevant text from manuscript
**Title and abstract**	1	(*a*) Indicate the study's design with a commonly used term in the title or the abstract	1	A cross-sectional study with 367 participants was conducted;
		(*b*) Provide in the abstract an informative and balanced summary of what was done and what was found	1	The study found a high prevalence of mothers….
**Introduction**				
Background/rationale	2	Explain the scientific background and rationale for the investigation being reported	2	In light of the significant concern related to the safety of breast milk sharing and to fully comprehend its health benefit and that of an HMB, it is vital to first understand mothers' knowledge and attitude towards HMBs.
Objectives	3	State specific objectives, including any prespecified hypotheses	3	A study was conducted to determine the motivators and barriers among mothers towards the acceptance of an HMB in Malaysia.
**Methods**				
Study design	4	Present key elements of study design early in the paper	4	A cross-sectional study via online surveys…
Setting	5	Describe the setting, locations, and relevant dates, including periods of recruitment, exposure, follow-up, and data collection	4	…. were conducted from June 2021 until November 2021. A self-administered validated questionnaire formatted as google forms were distributed via social media and WhatsApp

				targeting Malaysian mothers who were currently pregnant or those who were either breastfeeding or who had breastfed before were invited to participate.
Participants	6	(*a*) *Cohort study*—Give the eligibility criteria, and the sources and methods of selection of participants. Describe methods of follow-up<br>*Case-control study*—Give the eligibility criteria, and the sources and methods of case ascertainment and control selection. Give the rationale for the choice of cases and controls<br>*Cross-sectional study*—Give the eligibility criteria, and the sources and methods of selection of participants	4	The inclusion criteria were Malaysian mothers who were currently pregnant or mothers who were either breastfeeding or who had breastfed before, well versed in English and Malay, and those who provided consent. Non-Malaysian mothers, mothers who could not communicate in English or Malay, and mothers who did not provide consent were excluded. A convenient sampling technique was used. The participation information sheet and consent form were also available online, and each participant was required to fill out both forms prior to answering the questionnaire. Each participant was required to complete all the questions in the self-administered questionnaire.<br><br>The respondents were selected conveniently to be included in

				the analysis after fulfilling the inclusion and exclusion criteria.
		(*b*) *Cohort study*—For matched studies, give matching criteria and number of exposed and unexposed<br>*Case-control study*—For matched studies, give matching criteria and the number of controls per case		
Variables	7	Clearly define all outcomes, exposures, predictors, potential confounders, and effect modifiers. Give diagnostic criteria, if applicable	4, 5	The content of the online self-administered questionnaire was divided into to three domains, which included four parts. The domains of interest were sociodemographic factors, knowledge and attitude. Those participants who were willing to accept the establishment of an HMB in Malaysia were categorised as HMB acceptance and those who did not as HMB reluctance.
Data sources/ measurement	8*	For each variable of interest, give sources of data and details of methods of assessment (measurement). Describe comparability of assessment methods if there is more than one group	5	The independent variables included general knowledge of the benefits of breastfeeding, knowledge of breast milk sharing and HMBs, and attitudes towards breast milk sharing and HMBs. The dependent variable was the perceived acceptance of the establishment of an HMB in Malaysia. The background variables were age, religion, marital status, education level, employment status and household income. For HMB attitude and knowledge-related questions, correct and positive answers were given one mark for scoring without any negative marking involved. The total

				score for knowledge ranged from 0 to 18 and scores of more than 13 indicated good knowledge, while the total score for attitude ranged from 0 to 19 and scores of more than 10 indicated positive attitude towards HMB.
Bias	9	Describe any efforts to address potential sources of bias	18	Explained under limitation section
Study size	10	Explain how the study size was arrived at	4	Sample sizes were estimated based on a study by Keim et al (2014) on breast milk sharing attitude, whereby the lowest odds ratio was 3.43 for mothers who pumped milk to feed their child and the highest odds ratio was 3.7, which was the gestation age of the infant (26)

Continued on next page

Quantitative variables	11	Explain how quantitative variables were handled in the analyses. If applicable, describe which groupings were chosen and why	5	The completed Google questionnaires were exported from Microsoft Excel to SPSS for further analysis. The raw data were cleaned and coded accordingly for tabulation. Continuous data that were normally distributed were summarised in the mean and standard deviation, while categorical data were reported in frequencies and percentages. The independent variables were then determined for association with the acceptability to the establishment of an HMB using inferential analysis either by adopting Pearson's chi-square test or Fischer's exact test with statistical significance set at $p < 0.25$. Variables with $p < 0.25$ were then included in the multivariate binary logistic regression model to identify statistically significant predictor variables. The adjusted odds ratio (AOR) was computed, and $p < 0.05$ was statistically significant. The results were then presented as either tables or graphs.
Statistical methods	12	(*a*) Describe all statistical methods, including those used to control for confounding	5	As above (number 11)
		(*b*) Describe any methods used to examine subgroups and interactions	5	As above (number 11)
		(*c*) Explain how missing data were addressed		No possibility to have missing data as each participant have to fill up each question before proceeding to the next page in the google questionnaire.
		(*d*) *Cohort study*—If applicable, explain how loss to follow-up was addressed<br>*Case-control study*—If applicable, explain how matching of cases and controls was addressed		

		Cross-sectional study—If applicable, describe analytical methods taking account of sampling strategy		
		(*e*) Describe any sensitivity analyses		Not applicable
**Results**				
Participants	13*	(a) Report numbers of individuals at each stage of study—eg numbers potentially eligible, examined for eligibility, confirmed eligible, included in the study, completing follow-up, and analysed	6	A total of 367 respondents were included in the analysis, of whom all consented and completed the questionnaire, with no missing data.
		(b) Give reasons for non-participation at each stage		No missing data
		(c) Consider use of a flow diagram		
Descriptive data	14*	(a) Give characteristics of study participants (eg demographic, clinical, social) and information on exposures and potential confounders	6	The ages of the respondents ranged from 20 to 49 years old, with the majority being less than 35 years of age, as shown in Table 1. The average age of the respondents was 31.76 ± 4.44 years. The majority of the respondents were Muslim, had successfully completed their tertiary education, and were employed (73.3%, 82.8% and 70.8%, respectively). Furthermore, almost half of the respondents (51%) had a household income of ≥ RM 5000. The majority had satisfactory knowledge of the benefits of breastfeeding, with 257 out of 367 (70%) showing good scores (score 8 or more out of 10). This is shown in Tables 2.
		(b) Indicate number of participants with missing data for each variable of interest	6	No missing data
		(c) *Cohort study*—Summarise follow-up time (eg, average and total amount)		
Outcome data	15*	*Cohort study*—Report numbers of outcome events or summary measures over time		
		Case-control study—Report numbers in each exposure category, or summary measures of exposure		
		Cross-sectional study—Report numbers of outcome events or summary measures	5,13	HMB acceptance was the dependent variable in this study with those

				participants who were willing to accept the establishment of an HMB in Malaysia were categorised as HMB acceptance and those who did not as HMB reluctance. (Table 7)
Main results	16	(*a*) Give unadjusted estimates and, if applicable, confounder-adjusted estimates and their precision (eg, 95% confidence interval). Make clear which confounders were adjusted for and why they were included	13	Table 7: Association between the factors and respondent's agreement to the establishment of an HMB in Malaysia.
		(*b*) Report category boundaries when continuous variables were categorized		Not applicable
		(*c*) If relevant, consider translating estimates of relative risk into absolute risk for a meaningful time period		

Continued on next page

Other analyses	17	Report other analyses done—eg analyses of subgroups and interactions, and sensitivity analyses		Not applicable
**Discussion**				
Key results	18	Summarise key results with reference to study objectives	18	In conclusion, the present study has found a high prevalence rate of mothers (67.8%) who were willing to accept the establishment of an HMB in Malaysia. The main motivator towards the acceptability of an HMB was good knowledge of the benefit of breast milk and breastfeeding, while a significant barrier was related to religion reservation
Limitations	19	Discuss limitations of the study, taking into account sources of potential bias or imprecision. Discuss both direction and magnitude of any potential bias	18	However, the present study was based on each participant's self-perception at the given time. This perception may change over time, place and context and might produce a different result in a different setting. Selection bias was another limitation. The sampling technique used was convenient sampling because of the recent pandemic, which may have influenced the type of participants recruited. It should also be noted that many quantitative respondents were recruited from social media breastfeeding groups, affecting their level of knowledge and pre-existing confidence, making them more receptive to novel interventions like an HMB. Furthermore, mothers from rural areas and those without access to technology may not have been included because it may be difficult to capture their

				participation. Finally, there might also be a component of social desirability because the participants may be afraid of the stigma arising from their answer on this religiously sensitive topic; therefore, the participants were informed and given reassurance of their confidentiality and the level of anonymity.
Interpretation	20	Give a cautious overall interpretation of results considering objectives, limitations, multiplicity of analyses, results from similar studies, and other relevant evidence	14-17	Discussion section
Generalisability	21	Discuss the generalisability (external validity) of the study results	18	The study's ethnic composition is comparable to Malaysia's general ethnic structure (22), however, it is important to carefully interpret the results of this study to the general population with caution.
**Other information**				
Funding	22	Give the source of funding and the role of the funders for the present study and, if applicable, for the original study on which the present article is based	18	The author (s) received no specific funding for this work.

*Give information separately for cases and controls in case-control studies and, if applicable, for exposed and unexposed groups in cohort and cross-sectional studies.

Note: An Explanation and Elaboration article discusses each checklist item and gives methodological background and published examples of transparent reporting. The STROBE checklist is best used in conjunction with this article (freely available on the Web sites of PLoS Medicine at http://www.plosmedicine.org/, Annals of Internal Medicine at http://www.annals.org/, and Epidemiology at http://www.epidem.com/). Information on the STROBE Initiative is available at www.strobe-statement.org.
